# Diagnostic value of liquid biopsy in the era of precision medicine: 10 years of clinical evidence in cancer

**DOI:** 10.37349/etat.2023.00125

**Published:** 2023-02-28

**Authors:** Vincenza Caputo, Fortunato Ciardiello, Carminia Maria Della Corte, Giulia Martini, Teresa Troiani, Stefania Napolitano

**Affiliations:** Medical Oncology, Department of Precision Medicine, University of Campania “Luigi Vanvitelli”, 80131 Napoli, Italy; Nice University Hospital, France

**Keywords:** Liquid biopsy, circulating tumor DNA, precision medicine, clinical trials, minimal residual disease

## Abstract

Liquid biopsy is a diagnostic repeatable test, which in last years has emerged as a powerful tool for profiling cancer genomes in real-time with minimal invasiveness and tailoring oncological decision-making. It analyzes different blood-circulating biomarkers and circulating tumor DNA (ctDNA) is the preferred one. Nevertheless, tissue biopsy remains the gold standard for molecular evaluation of solid tumors whereas liquid biopsy is a complementary tool in many different clinical settings, such as treatment selection, monitoring treatment response, cancer clonal evolution, prognostic evaluation, as well as the detection of early disease and minimal residual disease (MRD). A wide number of technologies have been developed with the aim of increasing their sensitivity and specificity with acceptable costs. Moreover, several preclinical and clinical studies have been conducted to better understand liquid biopsy clinical utility. Anyway, several issues are still a limitation of its use such as false positive and negative results, results interpretation, and standardization of the panel tests. Although there has been rapid development of the research in these fields and recent advances in the clinical setting, many clinical trials and studies are still needed to make liquid biopsy an instrument of clinical routine. This review provides an overview of the current and future clinical applications and opening questions of liquid biopsy in different oncological settings, with particular attention to ctDNA liquid biopsy.

## Introduction

In the last decade, the concept of precision medicine in oncology has emerged as an innovative approach based on the ability to sequence each patient’s tumor. The precision medicine aims to optimize treatments and understand the dynamic evolution of cancer under therapeutic pressure [1–3].

Tissue biopsy is currently the standard method for diagnosis and molecular characterization of the tumors. However, it has many limitations due to its invasiveness and potential complications, such as bleeding, injury, infection, pain, tissue accessibility, and sample adequacy (such as insufficient amount and inadequate quality of the sample) [4].

In this context, the liquid biopsy has rapidly demonstrated its utility of being a non-invasive repeatable diagnostic test, able to profile the molecular asset of every single tumor and to guide the clinical management of cancer patients.

The U.S. National Cancer Institute (NCI) defines liquid biopsy as “a test done on a sample of blood to look for cancer cells from a tumor that are circulating in the blood or for pieces of DNA from tumor cells that are in the blood; a liquid biopsy may be used to help find cancer at an early stage which may also be used to help plan treatment or to find out how well treatment is working or if cancer has come back. Being able to take multiple samples of blood over time may also help doctors understand what kind of molecular changes are taking place in a tumor.” [5].

The wide and rapid diffusion of the liquid biopsy in different oncological clinical settings is due to the development of several different comprehensive genomic profile assays and a wide number of preclinical and clinical studies to better define the clinical utility of liquid biopsy [2, 6].

In the last years, many technologies and assays have been developed, many clinical applications have been investigated and different biomarkers have been discovered for the liquid biopsy test. Among these, the circulating tumor DNA (ctDNA) has emerged as a promising biomarker in the liquid biopsy test for clinical needs.

In this review, the current and future clinical applications of liquid biopsy for different solid tumors will be discussed, exploring the diagnostic value and the limitations of this test. This work aims to stimulate discussion and encourage further studies.

## Liquid biopsy

### History of liquid biopsy

For a long time, the effort of researchers and clinicians in the oncology field has been addressed to find sensible and specific cancer biomarkers or tests, allowing early cancer detection and better cancer management. The current biomarkers of liquid biopsy have been discovered many decades ago but only recently have been used in the management of cancer patients, maybe due to the limitations of available technologies.

As early as 1869, Ashworth [7] identified for the first time the circulating tumor cells (CTCs) in the metastatic cancer patient’s blood. In 1948, Mandel and Métais [8] recognized cell-free DNA (cfDNA) for the first time, but only in the 1970s, cfDNA was identified in cancer patients’ blood. In the 1960s, extracellular vesicles (EVs) were observed for the first time, but only in 2017, they were recognized as cancer biomarkers [9].

Even if they were discovered many decades ago, only in the last years they have captured the attention of scientific community as useful diagnostic tools, due to the diffusion of commercial and homemade liquid biopsy tests [10]. In fact, the development of sequencing technologies and the improvement of cancer genomic knowledge [11] lead to the diffusion of several comprehensive genomic profiling assays to personalize cancer patients’ management, promoting the diffusion of liquid biopsy.

**Figure 1.**
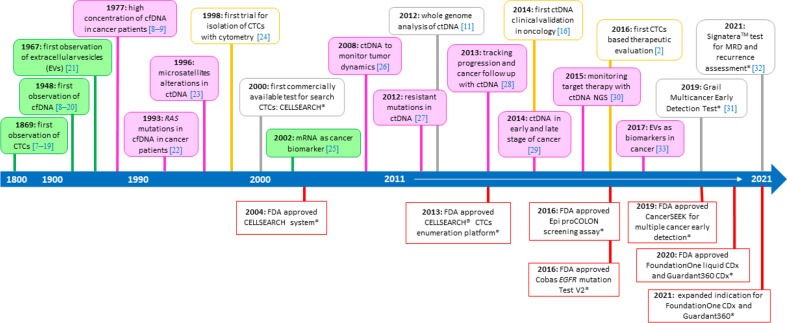
The timeline of liquid biopsy development. The figure illustrates some of the significant events in the development and spread of the liquid biopsy in clinical practice. * Some of the Food and Drug Administration (FDA) approved tests, addressed in the following text of the manuscript (“ctDNA liquid biopsy assays” and “Clinical applications of ctDNA liquid biopsy”); NGS: next generation sequencing; MRD: minimal residual disease

The concept of the liquid biopsy appears in the 1970s on Pubmed platform referred to the analysis of CTCs from the blood of patients affected by different solid tumors, but only in the last decade the number of articles about liquid biopsy has increased progressively, up to the present days when the literature about liquid biopsy is extremely wide (Figure 1) [12–33].

### Biomarkers of liquid biopsy

The term liquid biopsy is usually applied to blood samples mainly for the analysis of ctDNA and in part of CTCs, but many other biomarkers and types of samples are emerging [34].

The tumor microenvironment includes a mix of cellular and non-cellular components, many of which are released from cancer cells, which contribute to cancer survival, invasion, metastatic process, and resistance to treatments. Among these components, CTCs, ctDNA, cfDNA, EVs, or exosomes stood out for their characteristics as useful biomarkers circulating in the peripheral blood of cancer patients.

In blood samples, both in the plasma or serum, and in the cellular fraction, different circulating biomarkers can be detected and characterized [6].

In the plasma or serum, tumor-derived EVs or exosomes [35], proteins, circulating noncoding and messenger RNA [circulating free RNA (cfRNA)] [36], ctDNA [37], and tumor-educated platelets (TEP) [38, 39] can be detected. In the cellular fraction, single CTC and CTC clusters (constituted with CTCs exclusively or with CTCs escorted by immune cells) [40], circulating endothelial cells (CEC) [41], and cancer-associated (CA) fibroblasts can be identified [6]. Leukocytes and T-cell receptor profiling may also be potential biomarkers for metastatic patients treated with immunotherapy [42, 43].

The liquid biopsy biomarkers are present also in different body fluids, expanding the concept of liquid biopsy not only to the peripheral cancer patients’ blood but also to other fluids like prostatic fluid, urine (particularly for renal, bladder, and prostate cancers) [44], cerebrospinal fluid (CSF, particularly for central nervous system metastasis and primary brain cancers) [45], saliva or sputum, pleural effusion (particularly for head and heck and lung cancers) [46], tears, bile, peritoneal lavage, and bone marrow (particularly for breast, prostate, and lung cancers), stool, breast milk, and ascitic fluid [47–49].

### CTCs

The CTCs are cancer cells with a median half-life of 1–2.5 h, released in the blood from the primary tumor or metastatic sites at any stage of tumorigenesis [50]. They are present in the circulation as individual cells or in clusters (in this case, they have an increased metastatic ability). The CTCs have a high heterogeneity, according to their genomic, transcriptomic, proteomic, and metabolomic characteristics [51] and their phenotypic aspect depends on cancer type and stage of disease (usually associated with the worst prognosis) [52]. Their interactions with blood and tissue components are crucial for cancer survival and promotion of metastatic disease. The CTC blood concentration is very low and metastatic patients may have 1–10 CTCs per mL of blood [53].

Due to the very low concentration of CTCs, their analysis is difficult, expensive, and technically challenging. Complex technologies are needed to isolate and analyze CTCs and many platforms and assays have been developed for detecting CTCs through recognition of cell size, or expression of cell-surface antigens. In fact, CTC isolation requires different steps such as enrichment, detection, and cell characterization [54–57]. The enrichment step is based on the identification of their physical (size, elasticity, and density) and biological properties to increase CTC concentration. The detection step is based on immune-cytologic, molecular, or functional assays [54, 57, 58]. In the end, the CTC characterization is possible and CTC cultures, CTC lines [59–61] and CTC xenografts can be established [62–64]. There are several commercial assays for the enrichment, detection, and characterization of the CTCs. The CellSearch^®^ system is the most used and it is the only FDA-approved assay for searching CTCs [65]. The EPithelial ImmunoSPOT (EPISPOT) assay is also a clinical validated assay for many different cancers, including breast, prostate, head and neck cancers, and melanoma [66–68]. Other examples of CTC commercial assays for the liquid biopsy are Oncotype diagnosis (DX) androgen receptor splice variant 7 (AR-V7), oncotype sequencing (SEQ), Adnatest^®^, Epic Sciences, RareCyte, and EPithelial ImmunoSPOT in a DROP (EPIDROP). The CellSearch^®^ system was approved by FDA in 2004 to predict outcomes in metastatic breast cancers. In 2013, FDA expanded the indication of CellSearch^®^ CTC enumeration platform, to monitor patients with colon and prostate cancers [69–71]. In fact, the CTCs have been detected and studied in several different types of cancer, but their role as useful biomarker to predict outcomes, identify MRD, guide therapeutic choice, and monitor cancer progression is more evident in breast, prostate, colon, and lung cancer patients [40, 72–75].

The identification of CTCs allows the preservation of cellular contents and the expression of gene information, consenting functional analysis of CTC cultures, molecular characterization of genome, and protein analysis [76]. For example, the expression of AR-V7 splice variant on the CTCs of metastatic prostate cancer patients strongly predicts the resistance to abiraterone and enzalutamide [77]. Different studies have demonstrated that CTC’s functional and molecular characterization may have a prognostic value in cancer patients and may indicate the tumor’s capacity to become metastatic [53]. They are also associated with shorter progression-free survival (PFS) and overall survival (OS; metastatic breast, colorectal, and prostate cancers), as well as they may be used to monitor cancer evolution and response to therapy [78, 79]. In fact, the CTCs appeared to be generally more expressed in aggressive diseases [80, 81]. In addition to the prognostic value, their molecular characterization can provide additional information about the site of CTC origin (different organ microenvironments can select different types of CTCs), the ability to disseminate, the drug susceptibility or resistance in cell culture or xenograft, and the transcriptional plasticity [80–82].

Furthermore, the whole genome analysis (WGA) of a single CTC has shown great variability, confirming the tumor heterogeneity [83]. Based on these considerations, a blood sample may contain multiple cellular subpopulations and the analysis of a single CTC may not be representative of whole tumor biology [84].

However, CTCs are very difficult to detect due to the very complex and expensive method and the small number of cells in the blood at the early stage of diagnosis, during follow up and in the metastatic disease (often < 1 CTC per mL of blood). For these reasons, CTCs have limited clinical utility [85–89]. Further studies are needed to better understand the role of CTCs in the oncological precision medicine and detection technologies needed to be improved.

### Tumor-associated EVs

The tumor-derived EVs or exosomes are small round vesicles, generally 30–120 nm in diameter, delimited by a lipid bilayer that carries out proteins, DNA, RNA, small non-coding RNAs [microRNA (miRNA)], and lipids [53, 90, 91]. They are released by the tumor cells and used for communication between the tumor and other different cells [92, 93]. Each cancer cell type secretes a specific EV, allowing it to determinate the presence of the cancer and the cancer type [94, 95]. They are also found in abundant quantities in biological fluids, increasing the detection sensitivity [39, 93, 94]. The lack of unified adequate isolation methods and standardized analysis is a great limitation of EV implementation in the clinical setting [96]. The most diffused EV isolation technique is ultracentrifugation (UC), which separates EVs based on their size and density, and it requires dedicated equipment, excessive time, and costs [97]. The combination of different procedures, such as ultrafiltration and density gradient centrifugation, can increase the quality of EV isolated but can also reduce the amount of EVs [98–101]. In the last years, many commercial isolation kits have been developed trying to save time but with high costs [102].

Despite these limitations, EVs have been used as a novel biomarker in liquid biopsy for cancer monitoring, staging, and as a prognostic and dynamic tool for cancer therapy management [10, 93–95, 103–107]. However, the advantage of EV application to clinical settings is related to their various cargoes, allowing the identification of different and more specific molecular targets necessary for a personalized treatment [93–95]. The most common EV marker is the RNA and, in particular, the miRNA signature as predictive biomarker of prognosis and survival in different tumors, such as lung [108–110], liver [111, 112], colorectal [113], prostate [114], hepatocellular [115], breast [116], and pancreatic [117] cancers [10] and glioma [118]. For their nature and role, EVs can also be used to target tumor cells and to deliver and transfer drugs such as chemotherapies or immunotherapies in other cancer cells [10, 119–121]. For these reasons, recently, EVs are recognized as a promising potential liquid biopsy resource in oncology, but standardized and convenient methods for isolation are needed [91, 122].

The attention of the scientific community to the role of EVs as biomarkers for liquid biopsy in cancer is relatively recent, so further studies are needed to better understand their clinical utility in the oncological field.

### ctDNA

The DNA is released in fragments (cfDNA) through apoptosis, necrosis, and active secretion by normal (particularly lymphoid and myeloid cells, due to the frequent turnover of hematopoietic lineage cells of blood) [123], and cancer (ctDNA) cells [34, 37, 124, 125]. The concentration of ctDNA might only be < 0.01% of total cfDNA [58, 126]. The ctDNA is constituted of small DNA fragments (180–200 bp) released in the bloodstream of cancer patients. It has a short half-life (from 15 min to about 2.5 h) and a concentration range from 0 ng/mL to 1,000 ng/mL in blood (an average of 180 ng/mL in cancer patients) [125, 127, 128]. The fragment size of the ctDNA is variable, according to the different release mechanisms in the blood and the incomplete and random digestion of the ctDNA. The concentration and length of the ctDNA fragment can be a signature for prediction, diagnosis, and prognosis in cancer [129–133]. The ctDNA concentrations are higher in patients with metastatic cancers than those with localized cancers [134]. It is cleared through nucleosomes in the liver (liver macrophages), by circulating nucleases and in the kidney [124, 125, 129, 133, 134]. However, ctDNA shedding depends on several factors, like the tissue cell turnover (proliferation and apoptosis rate), the burden of disease, and the tumor site [124, 125, 129, 133, 134].

For its nature, the ctDNA provides information about point mutations, copy number variations (CNVs), structural rearrangements, loss of heterozygosity, gene fusions, methylation changes, integrated viral sequences associated with the tumor, and other genomic signatures [135–139].

The variant allele frequency (VAF) describes the proportion of ctDNA molecules containing a mutation over the total number of molecules containing the same allele [140–142].

The ctDNA recapitulates with accuracy the tumor characteristics and appears to be a diagnostic tool for many different solid tumors, for example, colorectal [143, 144], endometrial [145, 146], ovarian [78], breast [147], non-small cell lung cancer (NSCLC) [148–150], oropharyngeal [151], pancreatic [152], prostate [153, 154] cancers, and melanoma [155].

Currently, the ctDNA is the preferred biomarker for liquid biopsy analyses of druggable mutations. In fact, it can overcome costs, limitations and the difficulties in identification, extraction, and characterization of CTCs and EVs. Recently, a great number of technologies and commercial assays have been rapidly developed to detect ctDNA, and numerous studies have spread to investigate the potential role of this biomarker.

## ctDNA liquid biopsy assays

In the contest of ctDNA liquid biopsy, many different technologies and panel tests have been developed to analyze the molecular alterations for different purposes: polymerase chain reaction (PCR)-based sequencing can be used for single-locus/multiplexed assays and targeted sequencing for point mutations analysis, NGS-based sequencing can be used for single-locus/multiplexed assays, targeted sequencing, genome-wide analysis for point mutations, detection of rearrangements, chromosomal copy-number changes, and WGA [156–158].

The PCR methods use specific DNA probes to target specific known genes and they provide a quantitative measurement of the number of targets in the sample. They are highly sensitive and can detect a low tumor fraction DNA in plasma [159]. The NGS methods also use probes to capture specific DNA fragments and to target comprehensive known and unknown genes, but the data provide a ratio measurement and the sequences of the captured DNA. Moreover, a higher tumor DNA fraction than PCR is needed [142, 159].

Many authors have already examined different PCR and NGS techniques, so this review will make only a quick overview.

The real-time quantitative PCR (qPCR) is a fast and inexpensive tool [160] and it can detect mutant allele fraction (MAF) > 10% [161]. It is efficient when it analyzes a small number of variants, but it can assess only specified variant types, thus offering little discovery value [162].

The digital PCR (dPCR) is like qPCR in principle, but it is superior to qPCR in accuracy, although there are additional costs. It divides the sample into thousands of parallel PCR reactions, so the background noise is reduced. It is fast, inexpensive, and can detect MAF < 0.1% [163]. Multiplexed patient-specific panels can be used [164].

The droplet dPCR (ddPCR) is a type of dPCR. It genotypes a small number of known genes (also extremely infrequent mutations), interrogating multiple genes and at least 5–10 mutations simultaneously with a single ddPCR reaction. It is a sensitive, cost-effective, and fast technique. The ddPCR identifies and quantifies target DNA and provides results as absolute copies per mL. The ddPCR also offers little discovery value, because the number and types of targets are limited by assay design [162].

The dPCR has other different variants as beads, emulsion, amplification, and magnetics (BEAMing) [165]. It is extremely sensitive and has a detection rate of 0.02%, but it is technically complicated and relatively expensive for routine use [166].

The PCR-based assays are sensitive and convenient, and they are the most diffused assays in clinical practice, but they can only search for a limited number of known molecular alterations [157].

The NGS technology is based on massive parallel ultra-deep sequencing of millions of different DNA fragments in parallel, followed by computational analysis of reads, ensuring a high degree of sensitivity [16, 167]. The NGS is characterized by different steps: generation of a fragment DNA library, single fragment clonal amplification, massively parallel sequencing, and data analysis [168]. It has high throughput and it can search for many known and unknown multiple molecular alterations in multiple genes, involving single-nucleotide resolution of DNA sequences. Furthermore, it can discover new molecular alterations without prior knowledge at the end of the test, offering a higher discovery value [157]. Although it is more expensive than PCR, the comprehensive genomic profile nature of NGS can provide clinical knowledge (for its ability to cover more molecular targets) and cost when it is used for many patients at the same time [162]. The NGS detects MAF < 1% and many technical strategies are developed to increase sensitivity and reduce false negatives [169, 170].

Many commercial NGS tests are used to assess somatic alterations (including mutations, fusions, and copy number alterations), targeting simultaneously different genes. In fact, to improve sensitivity and detection power, the NGS methods have been applied to target panels, such as tagged-amplicon deep sequencing (TAm-Seq), safe-Seq system (Safe-SeqS), and cancer personalized profiling by deep-Seq (CAPP-Seq). The TAm-Seq identifies MAF of about 2% with a sensitivity > 97% [171]. The enhanced TAmSeq (eTAmSeq) can detect MAF of < 0.25% and it can identify single-nucleotide variants (SNVs), short insertions/deletions (indels), and CNVs [172]. The Safe-SeqS can reduce the sequencing errors about 70-fold and has a sensitivity of > 98% for detecting tumor mutations [173], while the CAPP-Seq detects MAF of < 0.02% and has a sensitivity of nearly 100% [174].

The whole-genome-Seq (WGS) is a non-targeted NGS technology, it searches for the whole genomic profile of the tumor DNA [159, 160, 175], providing a great amount of information. However, it is expensive and less sensitive and it requires a long time for the analysis. The whole-exome-Seq (WES), sequencing only the exons, is less expensive than WGS [176]. These two methodologies require high concentration of ctDNA. However, the WGA may be not convenient for cost, sensitivity, ctDNA amount, and necessary technologies. For these reasons, they have not yet found an application in clinical practice [157, 175, 176].

The PCR assays appear to be a good option on a large scale. They are the most diffuse technologies for high sensitivity and low cost. They are able to identify very low MAF of ctDNA, but for their nature of detecting only known point mutations, insertions and deletions, the knowledge about the tumor DNA is limited.

In the last years the NGS assays have had a huge spread, thanks to the increased sensitivity, diffusion of commercial companion diagnostic and agnostic panels (which can detect also low MAF of ctDNA), and the accessible costs [37, 142, 159]. The NGS is also applied to the untargeted panels, which do not need prior knowledge of molecular alterations, allowing to find genome-wide DNA variation [177, 178].

The possibility to search for a large number of actionable biomarkers and different known and unknown molecular alterations with a single test is essential for the development of precision medicine. The identification of gene alterations can be searched in both preclinical and clinical settings, allowing the development of new targeted therapies. The absence of standardization of several methods makes it difficult to compare different assays, therefore, it is not possible to define which is the better test to use in clinical practice. In fact, each test is useful for different cancer types and stage of disease and different purposes. In particular, the ctDNA concentration in plasma correlates with tumor size [133] and stage [179], therefore, patients with early cancer stages can have < 10 copies per 5 mL of tumor mutations *versus* patients with late cancer stages, in which the copies can be increased from 10 times to 100 times [180]. For this reason, in early cancer stages, the ctDNA assays need to be usually highly sensitive, while in late stages the sensitivity and the costs of ctDNA assays can be moderate. Therefore, the PCR assays may be useful for cancers with well-known genetic profiles, for molecular markers routinely searched and for diagnosis or monitoring the therapeutic response. The NGS panels may be useful for poorly defined or unknown origin cancers with an unexpected molecular profile with the aim to identify potential rare targeted alterations.

In the last years, many commercial tests have been developed, some examples are Archer^®^ Reveal ctDNA 28 (28 genes); OncoDNA OncoSTRAT&GO (27 genes); Guardant360^®^ companion diagnostic (CDx) [73 genes, microsatellite instability (MSI)]; Memorial Sloan Kettering-Analysis of Circulating cfDNA to Examine Somatic Status (MSK-ACCESS) (129 genes); FoundationOne^®^ Liquid CDx Assay [> 300 genes, blood TMB (bTMB), MSI, and tumor fraction]; and multiple other assays (Inivata InVision, AVENIO ctDNA panels, Oncomine Pan-Cancer cell-free Assay, Tempus xF Liquid Biopsy Assay, Signatera^TM^, UW-OncoPlex CT, elio Plasma Resolve, Therascreen*^®^*) [181]. In this context, the FDA approved some of them as companion diagnostic tests [182]. Examples of FDA-approved commercial tests for clinical application of ctDNA liquid biopsy are in Table 1 [182].

**Table 1.** Examples of FDA-approved commercial tests for clinical application of ctDNA liquid biopsy

**Test**	**Manufacturer**	**Cancer type indications**	**Biomarker (single cancer indication, pan-cancer/multi-cancer indications)**	**Technology**
Therascreen^®^, *PIK3CA RG*Q PCR Kit	Qiagen Manchester, Ltd.	Companion diagnostic Breast cancer: piqray (alpelisib)	*PIK3CA*	PCR
Cobas *EGF*R mutation Test v2	Roche Molecular Systems, Inc.	Companion diagnostic NSCLC: tarceva (erlotinib), tagrisso (osimertinib), iressa (gefitinib)	*EGFR*	PCR
Epi ProColon^®^	Epigenomics AG	Ancillary screening: colorectal cancer	*SEPT9* methylation	Bisulfite converted DNA and PCR
Guardant360^®^ CDx	Guardant Health, Inc.	Companion diagnostic NSCLC: tagrisso (osimertinib), rybrevant (amivantamab-vmjw), lumakras (sotorasib)	*EGFR*	NGS
FoundationOne^®^ Liquid CDx	Guardant Health, Inc.	Tumor mutation profiling: any solid tumor	73 genes	
FoundationOne^®^ Liquid CDx	Foundation Medicine, Inc.	Companion diagnostic NSCLC: iressa (gefitinib), tagrisso (osimertinib), tarceva (erlotinib), alecensa (alectinib), tabrecta (capmatinib) Metastatic castrate resistant prostate cancer (mCRPC): rubraca (rucaparib), lynparza (olaparib) Ovarian cancer: rubraca (rucaparib) Breast cancer: piqray (alpelisib)	*EGFR*, *ALK BRCA1/2*, *ATM BRCA1/2, PIK3C*A	NGS
		Tumor mutation profiling: any solid tumor	324 genes	
*BRAC* Analysis CDx	Myriad Genetic Laboratories, Inc.	Companion diagnostic Breast cancer: lynparza (olaparib), talzenna (talazoparib) Ovarian cancer: lynparza (olaparib), rubraca (rucaparib) Pancreatic cancer: lynparza (olaparib) mCRPC: lynparza (olaparib)	*BRCA*	PCR

The table illustrates the characteristics of some FDA-approved commercial tests as companion diagnostic tests and as ancillary screening tests for the ctDNA liquid biopsy. *PIK3CA*: phosphatidylinositol-4,5-bisphosphate 3-kinase catalytic subunit alpha; *RGQ*: rotor-gene Q; EGFR: epidermal growth factor receptor; *SEPT9*: septin 9; *ALK*: anaplastic lymphoma kinase; *BRCA*: breast cancer susceptibility genes; *ATM*: ataxia- telangiectasia mutated

## Strengths and weaknesses of ctDNA liquid biopsy

The molecular analysis of solid cancers alterations on formalin-fixed paraffin-embedded (FFPE) tissue has technical and sample availability challenges, such as longtime of processing, blindness of tumor heterogeneity, and limited amount or poor quality of samples, although sensitivity and specificity are excellent [183, 184].

The liquid biopsy assay is a minimally invasive option, with better sample quality and quick execution time, repeatable several times during the oncological history of patients, capturing spatial and temporal heterogeneity and clonal evolution of the cancer. Moreover, it has a high concordance rate with the tissue NGS [4, 183, 185]. A unique characteristic of the liquid biopsy is the possibility to perform the blood draw necessary for the analysis at the patient’s home [186]. It usually is technically faster than the tissue biopsy analysis and it can help to detect and genetically profile an occult malignancy in patients without tissue cancer available [187]. In addition, the ctDNA is more sensitive than the serum protein biomarkers [carcinoembryonic antigen (CEA), for example] [72].

However, the liquid biopsy increases costs, particularly when it is used concurrently with the tissue tests (it is often necessary when the analysis is negative). It is not able to analyze non-DNA biomarkers and it does not give histopathological and phenotypical information [such as programmed death-ligand 1 (PDL1) status] [4, 184]. Anyway, the liquid biopsy has also several limitations. First, the tumor signatures and actionable molecular alterations are not as frequent as it is expected. Furthermore, the ctDNA analysis requires high sensitivity and specificity to avoid false negative and false positive results. For these purposes, many technologies have rapidly developed, from commercial to homemade panels with different features, costs, and genomic information, underlying the need for standardized assays [7, 188–190].

Interestingly, the cancer and patient characteristics and the natural history of the tumor are important for the effectiveness of the liquid biopsy in the therapeutic choices. To reach these crucial needs, recently some expert recommendations for clinical use of ctDNA testing have been published, such as the European Society of Medical Oncology (ESMO) Scale for Actionability of Molecular Targets (ESCAT) and the OncoKB [191–193].

The false-positive and false-negative results are actually the major challenges that need to be addressed.

The false negative results are associated with a low percentage of mutated fragments below the limit of detection of the assay used. For example, a low tumor burden of disease, low or no ctDNA shedding due to the tumor site, vascularization or histology of the cancer lesions (such as the isolated brain metastasis), and rare allelic frequencies and variants limit the assay’s ability to detect mutations. The false negative results can also be given by a low sensitivity or by the absence of specific molecular alterations in the panel used (particularly in the small panels) [124, 125, 129, 133, 134, 159, 162, 172, 173, 194].

The false-positive results are associated with the sequencing errors, heterogeneity of tumor (for example, the difficulty to identify which clone dominates in which site), and background noises, like cfDNA shedding from cellular sources other than the tumor (for example, in elderly patient or patients with sepsis or inflammatory diseases) [195]. The molecular alterations from the cfDNA may originate from ctDNA, germline alterations, or non-tumor somatic alterations from white blood cells, such as clonal hematopoiesis of indeterminate potential (CHIP), that could not be identified in tumor biopsy [196–198]. In fact, the NGS liquid biopsy may be able to detect sequential mutational events in high turnover compartments, such as the bone marrow, which may not always reflect tumor genotype. These findings arise doubts regarding mutations detected in the cfDNA, especially in specific genes [for example, DNA methyltransferase 3 alpha (*DNMT3A*), additional sex combs like 1 (*ASXL1*), and ten-eleven translocation methylcytosine dioxygenase 2 (*TET2*), as well as tumour suppressor protein p53 (*TP53*), janus kinase 2 (*JAK2*), splicing factor 3B subunit 1 (*SF3B1*), guanine nucleotide-binding protein subunit beta-1 (*GNB1*), protein phosphatase magnesium-dependent 1 delta (*PPM1D*), guanine nucleotide-binding protein alpha stimulating (*GNAS*), and B-cell lymphoma (*BCL*) 6 corepressor-like 1 (*BCORL1*)] [196–198]. Determining the source of the cfDNA (tumor *versus* healthy tissue) remains a limitation of the cfDNA analysis. Finally, it must be considered that the identification of tissue from which cancer has raised, particularly for occult malignancy, is not always possible [187, 194].

The rapid growth of knowledge about genomic profile of different cancer types, the diffusion of many commercial panels and target therapies, and the big variability of information available have led to the rise of the multidisciplinary molecular tumor boards (MTBs) for overcoming the issues. The MTBs are groups of different specialists with expertise in different fields, with the purpose of interpreting the results obtained by the analysis of the ctDNA with liquid biopsy or the tissue NGS to support clinical application of these data [199]. Anyhow, further studies are needed to better understand how to integrate and interpret the liquid biopsy information in clinical practice.

## Clinical applications of ctDNA liquid biopsy

Although the tissue biopsy is actually the gold standard technique for cancer diagnosis and biomarkers evaluation, the use of the liquid biopsy has spread predominantly and rapidly in the management of patients with solid cancers at different stages of disease, integrating and improving the standard clinical care at different moments of patients’ cancer history. The advantage of profiling cancer genotypes without invasive procedures, particularly when the tumor tissue is insufficient or not available, makes the liquid biopsy an important tool in therapeutic decisions [200, 201]. The liquid biopsy is a minimally invasive and repeatable assay that can be used during longitudinal monitoring, making this test feasible for different aims, including screening. The ctDNA is the most investigated and used biomarker of the liquid biopsy in clinical practice applications [202–204]. It offers a snapshot of the genomic cancer profile, giving a real, instantaneous, and complete picture of the inter and intra-tumor heterogeneity [43, 202–205].

**Figure 2.**
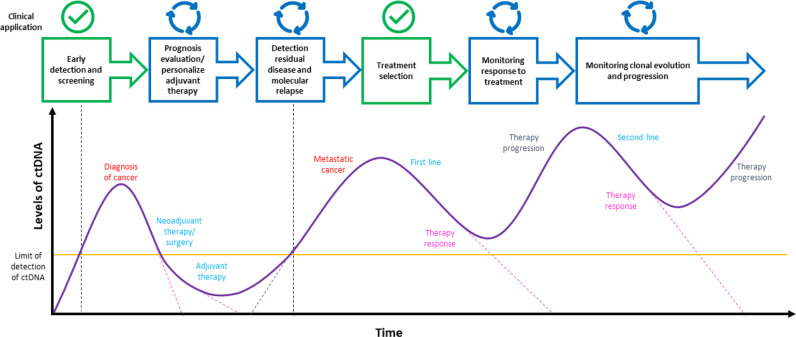
A schematic view of clinical applications of ctDNA liquid biopsy

There are many clinical applications of ctDNA liquid biopsy, some of which are widely applied and approved in clinical practice, others are under investigation and for all of them many works and clinical trials have been conducted and others are still in progress [202–204, 206–210]. Some clinical applications of the liquid biopsy are depicted in Figure 2, like the identification of cancer biomarkers and the detection of therapeutic targets for treatment selection [211], the real-time monitoring of therapeutic efficacy and response [212], the disease progression assessment and the identification of resistance mechanisms [213, 214], the early detection of cancer and the MRD assessment [58, 215], and the cancer screening [90, 216, 217].

### Treatment selection and resistant mechanisms

In the metastatic setting, the therapy decision-making process needs the genomic tumor analysis at the diagnosis and at the restaging of the disease [6]. In the targeted therapy era, the detection of druggable molecular alterations and the respective tailored treatment options can improve patient outcomes [218].

Despite the tissue biopsy is a gold standard technique for cancer diagnosis and molecular characterization, the liquid biopsy can be a complementary and even an alternative tool, considering a good concordance between the two methods and the high specificity and the moderate sensitivity of the latest one [174, 219–225]. For these reasons, the companion diagnostic tests have spread in the routinely clinical practice, particularly when the tissue is unavailable or insufficient (to search clonal alterations) and to detect acquired resistance mechanisms to therapy (to search clonal and subclonal alterations) [199, 226].

The NCI defines the companion diagnostic test as “a test used to help match a patient to a specific drug or therapy. For example, a companion diagnostic test may identify whether a patient’s tumor has a specific gene change or biomarker that is targeted by the drug. This helps determine if the patient should receive the drug or not. Companion diagnostic tests can also be used to find out whether serious side effects may occur from treatment or how well the treatment is working. Most drugs with a companion diagnostic test are cancer drugs that target specific tumor mutations.” [227]. Many guidelines have been developed to help clinicians in the use of the liquid biopsy, mainly for the interpretation of its results, such as ESMO recommendations and OncoKB recommendations [including FDA labeling, National Comprehensive Cancer Network (NCCN) guidelines, conference proceedings, disease-focused expert group recommendations, and the scientific literature] [181, 191, 193, 228].

The advanced NSCLC adenocarcinoma is the best example for the liquid biopsy application in molecular cancer profiling at the time of diagnosis [such as *EGFR*, *ALK*, c-ros oncogene 1 (*ROS1*), v-raf murine sarcoma viral oncogene homolog B1 (*BRAF*), *MET*, rearranged during transfection (*RET*) analysis)], when the tissue is insufficient for the molecular testing (about 50% of the cases) [181, 229] and a new tissue biopsy cannot be performed, or at the time of progressive disease after a targeted therapy with tyrosine kinase inhibitors (TKIs) [200, 201, 230]. For both purposes, the liquid biopsy has been integrated into the recommended guidelines and many companion diagnostic tests are available (such as Guardant360^®^, FoundationOne^®^ Liquid CDx, and Cobas). It has been shown that ctDNA assays can detect guideline-recommended biomarkers with a high concordance with the tissue analysis, and the power of detection increases when tissue and liquid biopsy are used complementary [163, 231]. The ctDNA can identify the acquired molecular resistances developed after treatment with EGFR TKIs (first or second generation), such as *EGFR* T790M mutation, and can guide the subsequent therapeutic choice (osimertinib) [232]. When osimertinib is prescribed in the first line therapy, *EGFR* C797S, *BRAF* V600E mutation, *MET* amplification or amplification or human epidermal growth factor receptor 2 (*HER2*) amplification can be detected in liquid biopsy as acquired resistance alterations [233–237]. In the metastatic *EGFR*-mutant patients, when the *EGFR*-positive ctDNA was detected after six weeks of osimertinib and bevacizumab treatment, it identified patients with high risk for early progression and lower OS [238]. The ctDNA analysis can also identify *ALK* fusion and related acquired resistance alterations, guiding the target therapies with TKIs [239], such as for *ROS1* [240] and *MET* molecular alterations [241]. Also, for ALK TKI therapies, the liquid biopsy may be a surrogate of outcomes, predicting the therapy response, according to the clearance of the blood ctDNA [242]. The emergence of *HER2* and Kirsten rat sarcoma (*KRAS*)*^G12C^* alterations powered the use of the liquid biopsy in the choice of the targeted therapy [241–245].

Anyway, if the liquid biopsy is negative (*EGFR* T790M, exon 19 deletion, and/or exon 21 L858R mutations and/or *ALK* fusion are not detected in ctDNA analysis, for example), a tissue testing is recommended [229, 246].

Furthermore, the NGS panels can also detect a plethora of unexpected molecular alterations of known or unknown meaning, that could give information about cancer evolution. Unfortunately, the NSCLC can switch under target therapeutic pressure to SCLC. In this scenario, the liquid biopsy is useless and the tissue biopsy is necessary [247].

In the HER2-negative and hormone receptor-positive (HER2–/HR+) metastatic breast cancer, the detection of *PIK3CA* mutations with the liquid biopsy can guide therapeutic choice with cyclin-dependent kinases 4/6 (CDK4/6) inhibitor alpelisib in combination with fulvestrant after endocrine therapy progression. Even in this case, when the ctDNA *PIK3CA* is negative, the tissue analysis is always necessary [248, 249]. The FDA approved as companion diagnostic test the Therascreen^®^, *PIK3CA RGQ* PCR Kit for the detection of the *PIK3CA* mutations with the liquid biopsy in breast cancer patients with this setting, based on the results of SOLAR-1 trial [249]. Furthermore, in *HER2* mutant non-amplified metastatic breast cancer patients treated with neratinib, the liquid biopsy in NGS demonstrated 100% specificity [250]. In the clinical trial plasma-based molecular profiling of advanced breast cancer to inform therapeutic choices (plasmaMATCH), the ctDNA was used for randomized patients in 5 cohorts based on mutation profiles [251]. In estrogen receptor-positive (ER+) metastatic breast cancer patients, the identification of estrogen receptor 1 (*ESR1*) and phosphatase and tensin homolog (*PTEN*) alterations by liquid biopsy is associated with the intrinsic resistance to aromatase inhibitors and alpelisib treatment [252].

For the metastatic colorectal cancer (mCRC) the NCCN guidelines do not directly address ctDNA tests and ESCAT guidelines consider the NGS only an alternative option to the PCR if it does not result in additional costs [228]. Despite this, the ctDNA was used for studying resistance mechanisms of cancer for the first time and showed high concordance with the tissue analysis [179, 253–256]. In the mCRC, the ctDNA liquid biopsy has shown its clinical utility in guiding first-line therapeutic choice by identifying rat sarcoma (*RAS*) mutations, which are negative biomarkers of response to anti-EGFR monoclonal antibodies therapy. The *KRAS* and neuroblastoma *RAS* viral oncogene homolog (*NRAS*) are also often acquired resistance alterations to target therapy and their identification is important for the re-challenge option with the anti-EGFR therapy in advanced lines [185, 255–259]. The *BRAF* V600E mutation detected by the ctDNA plays an important role in the choice of combined BRAF and EGFR inhibitors targeted therapy. This genomic profiling detection is important for the evaluation of acquired resistances to target therapy, too [260–262]. The mCRC is the best example to show the clinical utility of the ctDNA to identify and assess spatial and temporal heterogeneity in cancer [263]. In contrast to the tissue analysis, the ctDNA liquid biopsy gives an instantaneous collective snapshot of the cancer heterogeneity from all sites of cancer and at different time points in the history of a cancer patient [264–266]. An example of the cancer spatial heterogeneity of different metastatic sites is shown by the study of Parikh et al. [205] about mCRC patients, in which the post-progression ctDNA liquid biopsy captured all the acquired resistance alterations, that were later found separately in the distinct metastasis lesions by tissue biopsies. Siravegna et al. [267] showed how the ctDNA liquid biopsy can identify the different genomic evolution of each metastasis in mCRC, treated with HER2 blockade therapy [trastuzumab and lapatinib on the HER2 Amplification for Colo-rectaL cancer Enhanced Stratification (HERACLES) study]. They also showed how the ctDNA may help to understand the mixed radiological responses, highlighting the heterogeneity of anatomical response to the targeted therapy [261, 267]. An example of the cancer temporal heterogeneity [268, 269] is given by Parseghian et al. [270] study in which in the mCRC *RAS* wild type patients, after discontinuation of anti-EGFR antibodies, the ctDNA liquid biopsy predicts the decline of *KRAS* mutant-clones and provides evidence for re-challenge therapy with the anti-EGFR antibodies. Anyway, further studies are needed to validate the results of liquid biopsy as a tool to identify actionable molecular alterations to detect and manage the cancer’s spatial and temporal heterogeneity.

In 2020 the FDA approved the FondationOne liquid CDx as a companion diagnostic test for the use of rucaparib in *BRCA*-mutated mCRPC patients, demonstrating the feasibility and utility of the ctDNA in clinical practice also for this cancer type [182, 271].

In advanced melanoma, the liquid biopsy can improve the identification of *BRAF* and receptor tyrosine kinase (*KIT*) mutations for targeted therapy choice [272]. The *BRAF* V600 mutations were detected with the liquid biopsy in patients treated with BRAF and mitogen-activated protein kinase kinase (MEK) inhibitors before the radiological progression, with the lead time reduction of 110 days [273].

The PDL1 expression as a biomarker to select patients for immunotherapy remains limited for few cancer types and its determination requires tissue biopsy. The FDA approved pembrolizumab for genome signatures microsatellite instability-high (MSI-H) and high tumor mutational burden (TMB, ≥ 10 mut/Mb) in different tumors [274–276]. Some ctDNA assays can explore TMB and MSI status, overcoming the sampling bias and allowing pembrolizumab therapy with liquid biopsy analysis [206, 277, 278]. Particularly, the study that used ctDNA Guardant360^®^ assay to identify the MSI-H status showed clinical benefit from anti-PD(L)1 antibody therapy [277]. Wang et al. [279] have demonstrated that it is necessary a high-quality DNA to avoid underestimation of TMB for a correct clinical evaluation. Chauhan et al. [280] have detected and calculated the TMB from the ctDNA in urine rather than in blood [281]. Although many different guidelines (such as NCCN and ESMO guidelines) do not address plasma-based testing TMB or other tumor agnostic biomarkers like neurotrophic tyrosine receptor kinase (*NTRK*), the ctDNA analysis can help to choose the best treatment in selected cases [194].

Cancer resistance evolves dynamically under targeted therapy selective pressure and the ctDNA liquid biopsy can be a useful tool to detect these mechanisms of resistance [82].

### Treatment response monitoring and prognostic value

The usefulness of the ctDNA has been explored in many studies in different settings, including prognosis and treatment response monitoring [282–286]. The response monitoring using the ctDNA was born to track actionable oncogenic alterations during the targeted therapy. The feasibility of the ctDNA for this purpose became evident in different tumor types and in several settings [287, 288]. The monitoring of treatment response is also important to avoid ineffective therapies and unnecessary side effects. Moreover, many studies have analyzed the ctDNA value as a prognostic marker for clinical outcomes and as a tool for monitoring treatment response [289–291].

In the routine practice, the imaging is used to monitor treatment response sometimes in association with the tumor markers, unfortunately, their use is limited in sensitivity and specificity [58]. The ctDNA could be used as a personalized tumor-specific marker for patient surveillance and treatment efficacy monitoring [170, 270, 292]. Although the ctDNA has many issues, such as limited tumor shedding and burden of disease, the dynamic liquid biopsy can allow to monitor not only the tumor response but also the tumor evolution. Furthermore, in many scientific articles, the ctDNA decreasing or increasing clearance is associated with cancer response or progression to therapy respectively, with a shorter leading time than imaging [170, 270, 292, 293].

For example, in advanced *EGFR*-mutated NSCLC, early changes in the ctDNA value under targeted treatment with osimertinib providing efficacy information, particularly early clearance or decreasing levels of the ctDNA *EGFR* mutation (also known as molecular response) were associated with subsequent imaging response and with an improvement in response rates (RRs), PFS, and OS [294–296]. Conversely, higher ctDNA levels were associated with poor prognosis [297]. Several groups have demonstrated that early decreasing or clearance of the ctDNA in advanced NSCLC treated with immunotherapy or immunochemotherapy was associated with longer OS and a high RR, conversely increasing ctDNA, was correlated with the disease progression [170, 206, 298–300]. The ctDNA dynamics may also help to identify immunotherapy pseudoprogression, detecting a decrease of ctDNA levels *versus* increased tumor radiological volume, and conversely detecting an increase of ctDNA in true tumor progression [301]. Despite the diffusion of many works with similar results, further studies are needed to better investigate the role of the ctDNA as a biomarker for therapy response in different solid tumors.

In the metastatic breast cancer, the early ctDNA detection can predict response to palbociclib and fulvestrant therapy [302]. It can also predict therapy response and prognosis in the early breast cancer patients receiving neoadjuvant chemotherapy. In fact, the patients with positive ctDNA before neoadjuvant chemotherapy had significantly shorter disease-free survival (DFS) and OS than those with negative ctDNA [303].

In the advanced pancreatic cancer, a decrease of *KRAS* mutant ctDNA identified an earlier tumor response to the therapy, and it is strongly associated with clinical outcomes than blood levels of tumor markers [304, 305].

In a study by Lim et al. [306] conducted in *RAS* wild-type mCRC patients treated with chemotherapy and anti-EGFR antibody, the changes of the ctDNA VAF had linear correlation with tumor size and clinical outcome [307]. Moreover, in the *KRAS* mutant mCRC patients, the increasing ctDNA *KRAS* mutation allows to detect progression of disease earlier than imaging [308].

The dynamic ctDNA can correlate with outcomes and with radiological responses also in melanoma patients. Already in 2015, Gray et al. [309] evaluated the ctDNA to monitor treatment response in metastatic melanoma patients treated with targeted therapy or with immunotherapy. The patients who had persistently elevated ctDNA on both therapies had a poor prognosis; conversely, the decreasing or clearance of ctDNA at the first imaging follows up correlated with a better prognosis.

Anyway, the ctDNA has yet several limitations that must be overcome and many interventional clinical trials are needed to investigate its real clinical value, so the ctDNA is not actually used for monitoring treatment response or for prognostication in clinical practice and this remains an active challenge [310].

### MRD and risk assessment

The National Institutes of Health (NIH) defined the MRD as “a very small number of cancer cells that remain in the body during or after treatment. MRD can be found only by highly sensitive laboratory methods that are able to find one cancer cell among one million normal cells. Checking to see if there is MRD may help to plan treatment, find out how well treatment is working or if cancer has come back, or make a prognosis. MRD testing is used mostly for blood cancers such as lymphoma and leukemia, also called MRD.” [311].

Therefore, the MRD represents the detection of residual cancer cells (for example, ctDNA) without evidence of cancer disease at the conventional tests (for example, imaging, protein cancer biomarkers) during routine surveillance, after complete resection, neoadjuvant and/or adjuvant therapies [312]. If the cancer cells are still present after treatment, they may induce a relapse of disease. The MRD can help to classify patients with high or low risk of relapse and to predict short- or long-term relapse [313, 314]. The utility of the liquid biopsy to identify MRD is evident, considering the absence of other tools that can detect microscopic disease and its capacity to identify residual disease with a lead time of several months earlier than imaging. Moreover, the liquid biopsy can guide treatment adjustment and the surveillance during or after a curative treatment (for example, surgery and adjuvant chemotherapy) [315].

Although many different studies have investigated the clinical utility of the ctDNA to search the MRD, many limitations have been highlighted. For example, the MRD cannot be assessed by standard clinical and imaging technologies, the CHIP may be a confounding factor [316, 317], the timing of the ctDNA sampling between surgery and starting adjuvant therapy is often a crucial challenging point because it is difficult to choose the best moment (not too soon to detect the ctDNA set free from surgery, but not too late to start therapy) [318]. It is also very difficult to detect the ctDNA at a very low concentration with standard ctDNA non-personalized and uninformed assays, due to low sensitivity and lack of standardization of methodologies and technologies. Recently, many personalized informed technologies are developed to detect the MRD accurately, sequencing single tumor genotypes and designing a PCR or a NGS assay tailored to each patient. Tumor-informed ctDNA analysis is more sensitive than tumor naive analysis, but it is expensive and less feasible for clinical practice [310, 319, 320]. Examples of commercial panels to detect the MRD are Signatera^TM^ [Natera/Foundation Medicine (FMI)], Archer^®^Dx/Invitae-personalized cancer monitoring (PCM) assay, Guardant Reveal (LUNAR 1), Roche Avenio ctDNA surveillance kit, Inivata RaDaR/InVision MRD kit. Anyway, in a few years studies and clinical trials are increasingly trying to overcome these limitations and to select the best clinical and treatment approach in this new field [320–324].

Researchers have investigated the role of the ctDNA in the MRD not only for prognosis, but mainly for evaluated escalation or de-escalation treatments [325]. In patients with the MRD detected by the liquid biopsy after a curative treatment, an intensification or escalation treatment may be an optional strategy to improve DFS and OS. Conversely, in patients with the absence of the MRD after a curative treatment, a de-intensification or de-escalation therapy (such as omitting or depowering the adjuvant therapy schedule) can reduce side effects or toxicities [314, 326, 327].

In the NSCLC, adjuvant treatment has a modest survival benefit, thus the MRD can help to select the patients that really benefit from therapy [328, 329]. The TRACERx study has shown that the ctDNA MRD-positive predicted relapse before conventional imaging, with a median time of 164 days *versus* 362 days of imaging, with a significant lead time [330].

The DYNAMIC study evaluated the ctDNA dynamics after radical surgery, highlighting that the ctDNA MRD detected 3 days after surgery (R0) may be used as a baseline for lung cancer monitoring [331, 332]. The ctDNA can be used also in the unresectable locoregionally advanced NSCLC, to guide the escalation or de- escalation treatments. Moding et al. [333] have found that the detection of the ctDNA after chemoradiotherapy (CRT) or early on consolidation with the durvalumab was a strong predictor of risks of progression of disease. In contrast, the patients with the ctDNA not detected after CRT had better outcomes with or without consolidation immunotherapy, identifying patients in which therapy can be de-escalated [333]. A great number of studies are ongoing to better understanding where patients can benefit from adjuvant therapy and are cured by surgery alone, investigating the role of the ctDNA MRD in the management of the early-stage NSCLC.

The role of the ctDNA liquid biopsy has been evaluated for further different tumors. Garcia-Murillas et al. [334, 335] showed that, in the early breast cancer patients, the detection of the ctDNA after a curative therapy was associated with the metastatic recurrence, with a median lead time of 7.9 months over imaging and clinical recurrence.

Another study showed that in the ctDNA-positive muscle-invasive urothelial cancer patients, the adjuvant immunotherapy with atezolizumab can improve survival [336].

In the early CRC, a great number of studies have shown the utility of the ctDNA to detect the MRD and its role as a prognostic factor [337–341]. Reinert et al. [342] have shown that stage I–III CRC patients, with ctDNA- positive after a definitive therapy, had an increased risk of recurrence (about 40 times) than ctDNA negative patients during the follow-up. Notably, the lead time of the disease recurrence was 16.5 months (with respect to the imaging). Tie et al. [343] showed that, in locally advanced rectal cancer, the post-surgery ctDNA detection was strongly predictive of recurrence [344]. Tie et al. [345] also showed that in stage III CRC, the post-surgery ctDNA positive patients have a poor outcome, despite adjuvant therapy, with a 3-year-recurrence-free interval of 47% *versus* 76% in the ctDNA negative patients. Loupakis et al. [346] have evaluated the mCRC patients who had undergone metastatic resection with curative intent (PREDATOR clinical trial). The postsurgical MRD status was a strong prognostic biomarker associated with DFS. In fact, the MRD-positive patients had an inferior OS than the MRD-negative ones, also, the MRD-negative patients who did not receive systemic therapy had an OS of 100%.

The ctDNA has also been evaluated as a prognostic biomarker for the risk of the assessment in many different cancer types, as described above.

In another study, in the I–III stage NSCLC patients, the pre-surgery ctDNA detection identified patients at a higher risk of recurrence, which may reflect the micrometastatic disease [347].

Moreover, in the metastatic triple-negative breast cancer, the tumor fraction ≥ 10% was associated with a significantly worse survival [348].

In the metastatic *BRAF* V600 mutant melanoma, the *BRAF* ctDNA negative-patients at baseline therapy had a high RR to target therapy, while the *BRAF*-positive patients had a shorter PFS and OS [349].

Several studies have investigated the role of the ctDNA as prognostic biomarker, and many studies identified poor prognosis associated with high ctDNA levels.

Even if the ctDNA MRD may become a real-time biomarker for relapse of disease, many clinical questions are still open [210, 310, 350].

A great number of studies are ongoing in this field trying to delineate clinical utility of the liquid biopsy in the MRD.

### Early detection and cancer screening

The use of the ctDNA liquid biopsy in cancer early detection and screening is further investigated [351–355]. The liquid biopsy is a minimally invasive test, which can be repeated multiple times, and the blood draw is well accepted by people. However, in this setting further limitations arise due to the type of the population studied.

First, in the early detection and screening setting, a positive liquid biopsy identifies an “occult” cancer, with unknown origin, not detected by clinical and imaging standard. The detection of the CHIP mutations can give false positive results, limiting the specificity of the test in a setting in which high specificity is required high specificity. Furthermore, in the early cancer stage, the tumor burden is very low and consequently, the ctDNA levels are very low, giving false negative results due to the limited sensitivity of assays [330]. Additionally, some sensitive and specific tests are very expensive [356]. So, the liquid biopsy can over-diagnose or under-diagnose, or can just give an unclear clinical result, limiting its utility [310].

Multiple assays have been developed to implement the liquid biopsy as a good cancer screening test, such as the new ultrasensitive assays or using methylation markers.

Cohen et al. [357] have demonstrated the value of the liquid biopsy CancerSEEK platform for early detection of 8 different cancers, and its specificity was > 99% and sensitivity was 69–98% (all patients knew to have cancer).

The combination of different diagnostic approaches was investigated by Lennon et al. [216] in the Detecting Cancers Earlier Through Elective Mutation-Based Blood Collection and Testing (DETECT_A) study, using a multicancer test coupled with positron emission tomography/computed tomography (PET/CT) imaging to detect early cancer (no patient knew to have cancer), confirming the potential utility of this approach.

The ctDNA levels in the early-stage patients are lower than those in metastatic patients [330], and the sensitivity of a test is also given by both the blood volume sampled and by the number of mutations analyzed [358], so many commercial panels for early cancer screening probes multiple mutations [359].

Despite many limitations, the liquid biopsy can be a helpful tool for the cancer screening when other methods are not available, or patients refuse or are not fit for them.

The FDA has approved some liquid biopsy tests for the cancer screening, such as the Epi proColon^®^ for CRC, and have designed other liquid biopsy tests breakthrough device, such as the Grail’s Galleri, Natera’s Signatera^TM^, and CancerSEEK multicancer test, to support the current standard screening tests. The Epi proColon^®^ (Epigenomics) is a blood-based screening test, available for patients with an average risk of CRC, who have not completed the conventional guideline-recommended screening (unwilling or unable). It detects the tumor-associated epigenetic changes [360–362]. The CancerSEEK is a blood-based screening test; it identifies 8 different early-stage cancer types (breast, colon, esophagus, lung, liver, ovary, pancreas, and stomach), complementing other screening standard tools. It detects 8 biomarkers and mutations on 16 genes [363].

Furthermore, the liquid biopsy on urine may be useful for screening, and tests such as the UroSEEK or UroVysion have developed [364]. The UroSEEK detects mutations in 11 genes and aneuploidy for the identification of bladder and upper urothelial cancers. It has a sensitivity of 83% [365].

The early diagnosis remains a challenge, with sensitivity, specificity, and costs as critical factors. The liquid biopsy in this setting needs to overcome technical and clinical challenges. Further interventional and prospective, large-scale clinical trials are needed to demonstrate the clinical utility and the positive predictive value for cancer screening.

## Conclusions

In the era of precision medicine, it is well established that the cancer genomic profile is crucial to guide clinical decision-making. For this reason, the liquid biopsy assay has emerged as an innovative tool to guide the personalized approach for cancer patients, especially when the tissue samples are insufficient or limited in quality and quantity [366, 367]. The ctDNA analysis provides a real-time, non-invasive, and repeatable snapshot of the cancer molecular profile, offering several advantages in the diagnostic, prognostic field, and treatment selection. The wide diffusion of the ctDNA liquid biopsy is also due to the rapid development of commercial cost-effectiveness panel tests, available in the routinely clinical practice. Some evidence has demonstrated the attractive role of the ctDNA liquid biopsy in different clinical settings, such as early cancer detection, detection of MRD and early relapse, treatment selection, serial treatment response monitoring, prediction of outcome, risk assessment, and detection of resistance mechanisms. Actually, the ctDNA liquid biopsy is approved only for the treatment selection (companion diagnostic tests) and as an ancillary screening tool, many additional trials are needed to validate the clinical utility of this test in other settings. The future applications will be the therapy response assessment, the resistance monitoring, and MRD detection.

There is still to understand how to interpret the liquid biopsy results, as well as how to overcome the liquid biopsy clinical and technical limitations and the lack of technology standardization. The diffusion of new different fluids and biomarkers (EVs, miRNA), other than ctDNA and CTCs, and the development of patient personalized technologies for liquid biopsy could expand its applications. In fact, using them as complementary tools, each biomarker’s limitations could be partially overcome, moving from a genomic, transcriptomic, or proteomic evaluation to a multi-omic analysis of the cancer for better management of the patients. Furthermore, innovative clinical trials are ongoing, and many others will be designed to investigate the real clinical utility of the liquid biopsy and to integrate complementary information for the best clinical decision-making at any crucial moment of the cancer disease. Anyway, it is undoubted that the liquid biopsy is transforming the precision medicine in oncology as a promising revolutionary weapon for real-life oncology in the near future.

## Abbreviations

*ALK*: anaplastic lymphoma kinase
*BRCA*: breast cancer susceptibility genes
CDx: companion diagnostic
cfDNA: cell-free DNA
CHIP: clonal hematopoiesis of indeterminate potential
CTCs: circulating tumor cells
ctDNA: circulating tumor DNA
ddPCR: droplet digital polymerase chain reaction
DFS: disease-free survival
dPCR: digital polymerase chain reaction
EGFR: epidermal growth factor receptor
ESMO: European Society of Medical Oncology
EVs: extracellular vesicles
FDA: Food and Drug Administration
HER2: human epidermal growth factor receptor 2
*KRAS*: Kirsten rat sarcoma
MAF: mutant allele fraction
mCRPC: metastatic castrate-resistant prostate cancer
miRNA: microRNA
MRD: minimal residual disease
MSI: microsatellite instability
NCCN: National Comprehensive Cancer Network
NGS: next generation sequencing
NSCLC: non-small cell lung cancer
OS: overall survival
PCR: polymerase chain reaction
PFS: progression-free survival
*PIK3CA*: phosphatidylinositol-4,5-bisphosphate 3-kinase catalytic subunit alpha
*RAS*: rat sarcoma
RRs: response rates
TAM-Seq: tagged-amplicon deep sequencing
TKIs: tyrosine kinase inhibitors
TMB: tumor mutational burden
WGA: whole genome analysis

## Declarations

### Copyright

© The Author(s) 2023.
